# Case report of unusual synchronous anal and rectal squamous cell carcinoma: clinical and therapeutic lesson

**DOI:** 10.3389/fonc.2023.1187623

**Published:** 2023-06-08

**Authors:** Davide Ciardiello, Sara Del Tufo, Paola Parente, Antonietta Gerarda Gravina, Francesco Selvaggi, Iacopo Panarese, Renato Franco, Michele Caterino, Giulia Martini, Fortunato Ciardiello, Roberto Grassi, Salvatore Cappabianca, Alfonso Reginelli, Erika Martinelli

**Affiliations:** ^1^ Division of Gastrointestinal Medical Oncology and Neuroendocrine Tumors, European Institute of Oncology (IEO), IRCCS, Milan, Italy; ^2^ Oncology Unit, Department of Precision Medicine, Università degli Studi della Campania “Luigi Vanvitelli”, Napoli, Italy; ^3^ Radiology Unit, Department of Precision Medicine, Università degli Studi della Campania “Luigi Vanvitelli”, Napoli, Italy; ^4^ Pathology Unit, Fondazione IRCCS Casa Sollievo Della Sofferenza, San Giovanni Rotondo, Foggia, Italy; ^5^ Gastroenterology Unit, Department of Precision Medicine, Università degli Studi della Campania “Luigi Vanvitelli”, Napoli, Italy; ^6^ Department of Advanced Medical and Surgical Sciences, Università degli Studi della Campania “Luigi Vanvitelli”, Napoli, Italy; ^7^ Pathology UniV, Department of Mental and Physical Health and Preventive Medicine, University of Campania “Luigi Vanvitelli”, Napoli, Italy; ^8^ Clinica Villa delle Querce, Napoli, Italy

**Keywords:** SCC, rectal cancer, anal cancer, HPV, chemo-radiotherapy

## Abstract

Synchronous tumors of the rectum and anus are sporadic. Most cases in the literature are rectal adenocarcinomas with concomitant anal squamous cell carcinoma. To date, only two cases of concomitant squamous cell carcinomas of the rectum and anus are reported, and both were treated with up-front surgery and received abdominoperineal resection with colostomy. Here, we report the first case in the literature of a patient with synchronous HPV-positive squamous cell carcinoma of the rectum and anus treated with definitive chemoradiotherapy with curative intent. The clinical-radiological evaluation demonstrated complete tumor regression. After 2 years of follow-up, no evidence of recurrence was observed.

## Background

Squamous cell carcinoma (SCC) of the rectum is an infrequent malignancy. Only 0.1%–0.3% of rectal cancers (RCs) are represented by the SCC histotype, while adenocarcinoma represents about 90% of RCs (1, 2). Similarly, anal cancer is rare, accounting for less than 1% of all new cancer diagnoses and less than 3% of all gastrointestinal tract tumors (3). Synchronous tumors of the rectum and anus are sporadic. Most cases in the literature are rectal adenocarcinomas with concomitant anal SCC. Thus, identification of the optimal treatment in this unusual presentation is challenging and has to be defined case by case. To date, only two cases of synchronous SCC of the rectum and anus are reported, and both were treated with up-front surgery (4, 5). Here, we report the first case in the literature of a patient with synchronous SCC of the rectum and anus treated with definitive chemo-radiotherapy (CRT) without subjecting her to radical surgery.

## Clinical presentation

A 68-year-old woman came to our observation for the onset of pain in the anal area, weight loss, and nonspecific abdominal pain for about 2 months. The patient, a former smoker and nondrinker, presented in good condition with a performance status (PS) of 0 according to ECOG and without comorbidities. Blood test values were within the range. About a month before, following the indication of a general practitioner, the patient underwent an ultrasound exam of the abdomen, which resulted in a negative, and a colonoscopy. The endoscopic examination showed the presence of two lesions: a polyp of approximately 4 cm about 10 cm from the anal verge and another ulcerated lesion at the anorectal junction. Biopsies of both lesions were performed. The histological examination, conducted in a local laboratory, revealed in both cases nonkeratinizing SCC. No evidence of gynecological tumors was clinically observed.

Due to the unusual endoscopic presentation and histologic report, the case was discussed by a multidisciplinary team to define the best diagnostic and therapeutic flow.

**Figure 1:**
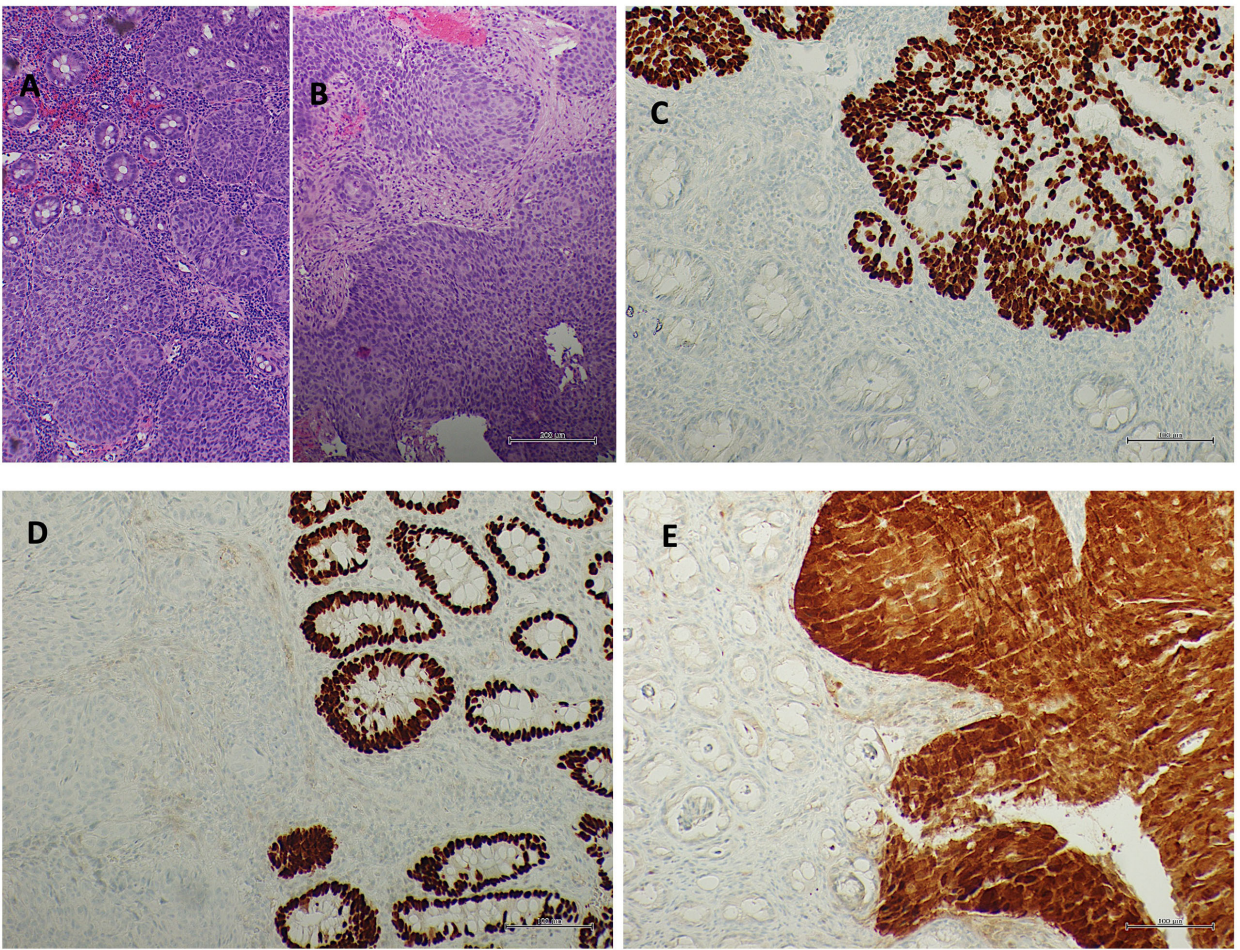
Rectal mucosa (**A** hematoxylin/eosin staining) and anal mucosa (**B** hematoxylin/eosin staining) infiltrated by squamous non-keratinizing carcinoma. Squamous differentiation underlined from p40 immunostaining (**C** p40 immunohistochemistry) and negativity for CDX2 (**D** CDX2 immunohistochemistry). Diffuse p16 immunostaining in neoplasia (**E** p16 immunohistochemistry).

It was decided to repeat the endoscopic examination and revise the tumor samples. The pan-coloscopy showed the presence at the level of the anorectal junction of an ulcerated lesion of approximately 15–20 mm and, about 8 cm from the anal verge, a lesion of about 3 cm with a nonlifting sign. A re-biopsy of each lesion was performed. At the microscopical examination, both rectal and anal biopsies confirmed the diagnosis, documenting infiltration by carcinoma with a solid growth pattern. Immunohistochemistry documented positivity for p40 and CK5/6 and negativity for CK20 and CDX2, leading to squamous, nonkeratinizing histotype, according to the WHO 2019 edition of Digestive System Tumors. Moreover, diffuse p16 immunostaining was shown, as observed in human papillomavirus (HPV) infection (**Figure 1**).

A baseline total body CT scan with the administration of a contrast medium documented the presence of a polypoid lesion at the level of the rectum, at the right posterolateral wall, and at the level of the anal region, where there was pathological thickening.

The MRI examination of the rectum showed the presence of a parietal formation with a polypoid appearance and a broad implant base at the level of the mid-rectum, measuring roughly 37 × 37 × 37 mm (AP × LL × CC) of the posterolateral wall right, causing narrowing of the lumen. This formation showed a restriction of the signal in the Diffusion-weighted imaging (DWI)/ADC sequences and clear pathological impregnation in the post-contrastography phases, infiltrating the mesorectum until it exceeds the mesorectal fascia by about 4.5 mm, determining the extramural vascular invasion and the elevator muscle of the right anus. At the level of the anorectal junction, the presence of heteroplastic tissue with dimensions of approximately 18 × 13 × 23 mm (AP × LL × CC) was highlighted, which infiltrates the internal and external anal sphincter, showing inhomogeneous intensity in T2 and signal restriction in DWI. Furthermore, two lymph node formations were found in the right posterolateral mesorectal fat, one 13 mm from the mesorectal fascia and another in the coccygeal area. Thus, both endoscopic examination and MRI demonstrated no contiguity between the two lesions. Therefore, it was not possible to define whether the two lesions have a common origin or whether they are two distinct neoplasms According to MRI evaluation, rectal cancer staging was T4b N1b CMR+ MVI+, while anal cancer staging was T2 N1a.

The multidisciplinary group discussed the case again to define the therapeutic program. Considering that chemoradiotherapy is a standard of care (SOC) for SCC of the anus and that available evidence shows that rectal SCC is also sensitive to this treatment, it was decided to propose concurrent chemoradiotherapy, reserving the option of surgery in the presence of persistence or locoregional progression (1–3).

Thus, the patient started treatment with mitomycin *c* 10 mg/m^2^ on days 1–29 scheme plus capecitabine 825 mg/m^2^ bis in die (bid) and concomitant radiotherapy on the pelvis and anorectum (total dose, 60 Gy).

**Figure 2:**
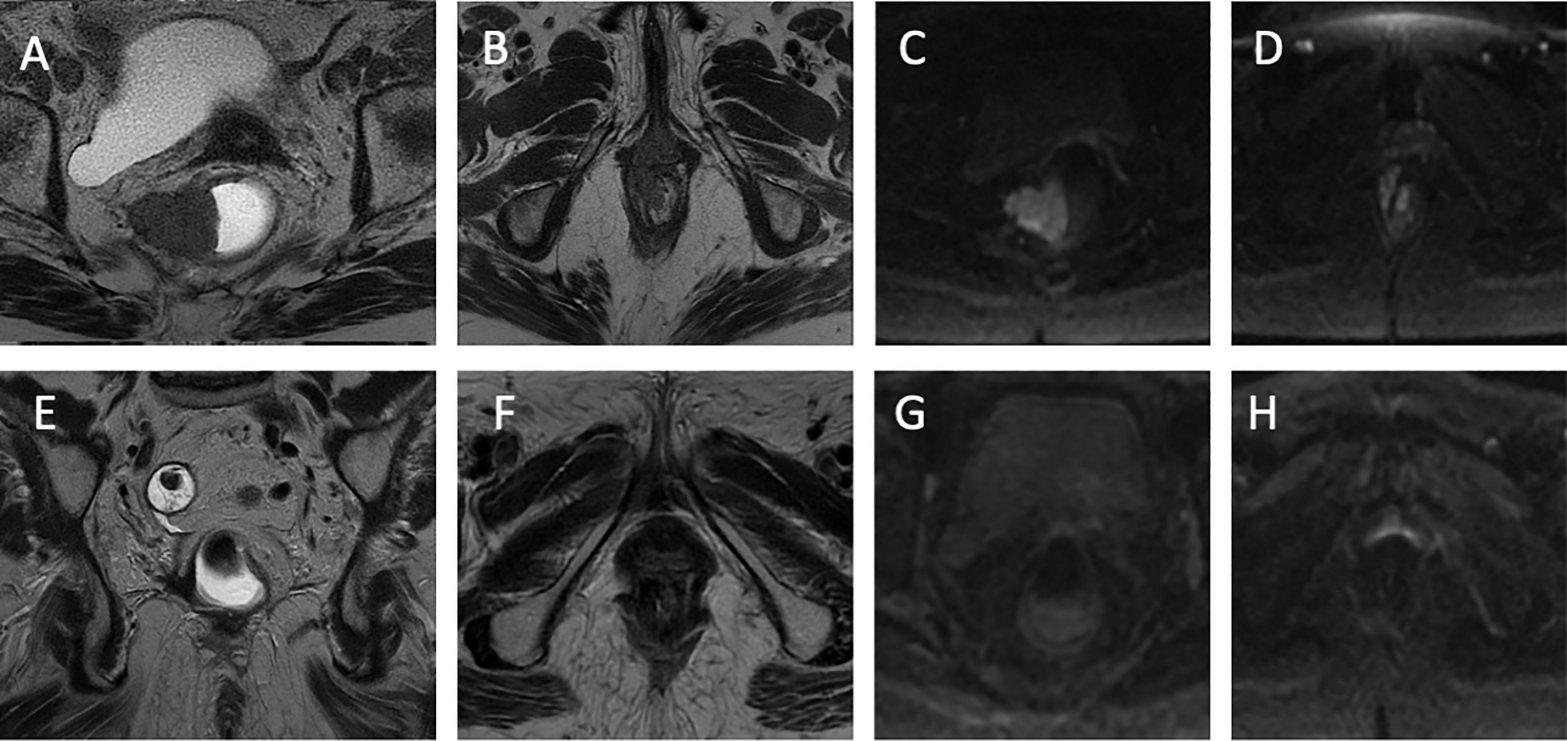
Radiologic images. **(A)** The T2-weighted sequence of rectal cancer on the axial plane. Parietal neoplastic formation of the middle rectum with a polypoid appearance and a broad implant base is observed on the right posterolateral wall from 6 to 12 o’clock, which causes the narrowing of the rectal lumen. This rectal formation infiltrates the mesorectum until it exceeds the mesorectal fascia by about 4.5 mm and infiltrates the elevator muscle. **(B)** The T2-weighted sequence of anal cancer on the axial plane. At the level of the rectal junction at 11 o’clock, there is evidence of neoplastic tissue infiltrating the internal and external anal sphincter. **(C)** Sequence weighted in DWI in correspondence with the rectal lesion. Signal restriction in the DWI sequence. **(D)** Sequence weighted in DWI at the level of the anal canal. Signal restriction in the DWI sequence. **(E)** Coronal plane T2-weighted sequence. A clear hypointensity is observed with regard to fibrotic outcomes 6 months after the start of radiochemotherapy (CRT). **(F)** T2-weighted sequence on the axial plane. Anal lesion response after CRT. **(G)** Sequence weighted in DWI in correspondence with the rectal lesion. No signal restriction following CRT. **(H)** Sequence weighted in DWI at the level of the anal canal. No signal restriction following CRT.

During therapy, the patient experienced grade 3 diarrhea and grade 2 anal mucositis, which required suspension of the concomitant therapy for 1 week, and symptomatic treatment for diarrhea and anal mucositis was administrated. After regression to grade 1 toxicity, the chemoradiotherapy treatment was continued. Response to treatment was evaluated with clinical, endoscopic, and instrumental criteria after 6 months from the beginning of chemoradiation. A digital rectal examination showed no evidence of disease, as did an endoscopic evaluation. Biopsies were taken during proctoscopy and were negative. Finally, re-evaluation with contrast-enhanced MRI examination showed complete tumor regression with no sign of a viable tumor in the DWI sequence (**Figure 2**). After 2 years of follow-up performed according to the European Society of Medical Oncology (ESMO) guidelines for anal cancer, no evidence of disease was observed (3). The patient maintained a good rectal function after CRT without an impact on her daily life.

## Discussion

The insurgence of SCC in the lower gastrointestinal tract is rare, with most of the tumors originating from the squamous epithelium of the anal canal (2). Primary SCC from the colon and rectum are rare, representing less than 1% of colorectal malignancies (1–5). So far, while different theories have been proposed, the etiology of rectal SCC is still debated (1, 2). It has been suggested that rectal SCC could originate from pluripotent stem cells that could differentiate into different lineages (6). Other groups suggested a potential malignant evolution from persistent ectopic embryonal nests of ectodermal cells (7). The presence of chronic inflammation such as intestinal bowel disease (IBD) that causes a persistent irritative stimulus could induce squamous metaplasia and favor the insurgence of rectal SCC (8). HPV and human immunodeficiency virus (HIV) are recognized risk factors for anal cancer (9). Nevertheless, the role of HPV infection in rectal SCC is controversial (10–12). Audeau and colleagues evaluated the association of HPV in a cohort of 20 patients with SCC of the rectum, adenosquamous tumors, or adenocarcinoma with squamous dysplasia; the authors reported no correlation with HPV 6, 11, 16, and 18 (10). On the contrary, other case reports or case series found a correlation with HPV positivity in squamous rectal cancer (11, 12).

Guerra and colleagues conducted a retrospective analysis on the Surveillance Epidemiology and End Results Database (SEER) to investigate the clinical–pathological characteristics of rectal SCC (13). In a large population of 142 patients diagnosed between 1946 and 2015, the median age was 63, with a predominance in women and in diagnosis in the early stage compared with advanced disease. The presence of synchronous rectal and anal SCC is an uncommon condition, and to date, only two cases were described in the literature (4, 5). The first one was a 48-year-old man with an anal and a rectosigmoid SCC with type 2 diabetes as the only comorbidity; no history of smoking or alcohol consumption was described (4). HPV was not tested. The patient underwent abdominoperineal resection, and a permanent colostomy was positioned. Subsequent adjuvant chemoradiotherapy was performed.

The second case was a 78-year-old man with a concurrent anal canal SCC and a rectal SCC (5). The patients had an anamnesis of heavy smoking, alcohol drinking, and opium consumption. No comorbidity or viral infection was reported. While the endoscopic and radiological evaluation demonstrated the presence of rectal and anal lesions, the biopsy results were negative. Thus, the patients underwent up-front diagnostic and therapeutic surgery with an abdominal–perineal resection. Histopathology proved the presence of synchronous rectal and anal SCC. After a multidisciplinary discussion, postoperative chemoradiotherapy was proposed.

In this scenario, our case could be of interest in different aspects. It represents the first case of concomitant HPV-positive rectal anal SCC described in the literature. It is very difficult to assess if the rectal SCC was a metastasis of anal cancer or a second malignancy. Repeated endoscopy evaluation, CT scan, and high-quality RMI do not demonstrate a clear contiguity between the two lesions. Moreover, from a clinical point of view, it is intricate to correlate a small anal cancer with a significantly more advanced rectal SCC. However, in both lesions, the presence of an HPV infection could have clearly contributed to the pathogenesis. Unfortunately, like in the other two cases, due to the lack of an adequate tumor sample, it was not possible to perform a genetic evaluation to discriminate if the two lesions have a common or distinct origin or to evaluate HPV genetic typing. This aspect could represent a limitation that deserves to be investigated by further translational prospective studies/case series. In the last decades, definitive CRT has emerged as the SOC for early and locally advanced anal SCC with curative intent (3). In cases of persistent disease or locoregional recurrent disease, surgery could represent a therapeutic option. Due to its infrequent occurrence, there has been no prospective study investigating the optimal treatment for rectal SCC (1). Historically, up-front surgery was proposed; however, it was complicated by significant comorbidity and mortality (1, 14). Therefore, definitive CRT has been proposed to improve outcomes and preserve organs (1, 15, 16). A French retrospective study included 23 patients treated in two referral institutions. CRT exhibited a really high rate of clinical complete response at 83%. The 5-year disease-free survival rate was 81%, while the 5-year overall survival rate was 86%. Remarkably, the 5-year colostomy-free survival rate was 65%. In another series of nine patients with locally advanced or metastatic rectal SCC, induction with docetaxel, 5-fluorouracil, and cisplatin (DCF) determined a promising response rate that was further increased after chemoradiation (16). For patients with metastatic disease, no evidence based on prospective studies is currently available. In a case series from the Mayo Clinic, 52 patients with advanced rectal SCC were included; however, the exact number of cases with metastatic disease was not indicated (17). Based on these findings, it is reasonable to treat rectal SCC similarly to anal SCC with CRT in cases of locally advanced disease and platinum-based chemotherapy in combination with 5-fluorouracil or taxane for metastatic disease (1).

Intriguingly, our case report is the first one to use a conservative approach for synchronous rectal and anal lesions. The patient received the combination of mitomycin *c* 10 mg/m^2^ on days 1 and 29 together with capecitabine 825 mg/m^2^ (bid) with concurrent radiotherapy for a total dose of 60 Gy with curative intent. According to the ESMO guidelines, while the optimal dose for curative CRT is not known, for patients with locally advanced anal cancer, the radiotherapy dose should be >50.4 Gy (3). In the absence of prospective studies, we can consider these recommendations also valid for rectal SCC. The definition of the best time for tumor assessment in rectal SCC is not yet defined. In the ACT II study, it has been shown that a significant proportion of patients with anal SCC treated with CRT do not exhibit a complete response when assessed at 10–12 weeks and could display a complete tumor regression at 26 weeks from the beginning of CRT (18).

Clinical and radiological evaluation after 6 months of the beginning of chemoradiation showed no evidence of disease and a complete clinical response. After a longer follow-up of 2 years, no evidence of occurrence was observed without residual toxicity.

## Conclusion

Rectal SCC is an uncommon malignancy with limited evidence to guide treatment decisions. In this scenario, we report the first case of synchronous rectal and anal HPV SCC treated with conservative CRT. While more cases are needed to better understand the biology and multidisciplinary approach, we think that our case report could be of interest in this orphan disease.

## Patient perspective

When I started my oncological journey, I was full of fears. I met doctors who helped and supported me through the hardest of times. Thanks to teamwork, more than 2 years after the diagnosis, I recovered and went back to living normally.

## Data availability statement

The original contributions presented in the study are included in the article/supplementary material. Further inquiries can be directed to the corresponding author.

## Ethics statement

Written informed consent was obtained from the individual(s), and minor(s)’ legal guardian/next of kin, for the publication of any potentially identifiable images or data included in this article.

## Author contributions

Conceptualization: DC. Original writing: DC, ST, and PP. Data collection: all the authors. Supervision: RG, SC, FC, AR, and EM. All authors contributed to the article and approved the submitted version.
